# Transfer Entropy Modeling of Newborn Cardiorespiratory Regulation

**DOI:** 10.3389/fphys.2020.01095

**Published:** 2020-08-27

**Authors:** Maristella Lucchini, Nicolò Pini, Nina Burtchen, Maria G. Signorini, William P. Fifer

**Affiliations:** ^1^Department of Psychiatry, Columbia University Irving Medical Center, New York, NY, United States; ^2^Division of Developmental Neuroscience, New York State Psychiatric Institute, New York, NY, United States; ^3^Dipartimento di Elettronica, Informazione e Bioingegneria (DEIB), Politecnico di Milano, Milan, Italy

**Keywords:** Transfer Entropy, cardiorespiratory regulation, multivariate modeling, sleep regulation, autonomic nervous system, prematurity

## Abstract

This study investigates the complex interplay between the cardiac and respiratory systems in 268 healthy neonates born between 35 and 40 weeks of gestation. The aim is to provide a comprehensive description of the developing cardiorespiratory information transfer mechanisms as a function of gestational age (GA). This report proposes an extension of the traditional Transfer Entropy measure (*TE*), which employs multiple lagged versions of the time series of the intervals between two successive R waves of the QRS signal on the electrocardiogram (*RR* series) and respiration time series (*RESP*). The method aims to quantify the instantaneous and delayed effects between the two processes within a fine-grained time scale. Firstly, lagged *TE* was validated on a simulated dataset. Subsequently, lagged *TE* was employed on newborn cardiorespiratory data. Results indicate a progressive increase in information transfer as a function of gestational age, as well as significant differences in terms of instantaneous and delayed interactions between the cardiac and the respiratory system when comparing the two *TE* directionalities (*RR→RESP* vs. *RESP→RR*). The proposed investigation addresses the role of the different autonomic nervous system (ANS) branches involved in the cardiorespiratory system, since the sympathetic and parasympathetic branches operate at different time scales. Our results allow to infer that the two *TE* directionalities are uniquely and differently modulated by both branches of the ANS. *TE* adds an original quantitative tool to understanding cardiorespiratory imbalance in early infancy.

## Introduction

Premature birth and related complications are the leading cause of death under 5 years of age across the world (Liu et al., 2016). According to the March of Dimes, in the United States, the percentage rate of preterm birth in 2019 was 10.00%, marking the third consecutive year of increase after 7 years of decline (March of Dimes, 2019). Epidemiological studies have shown that late preterm [LPT: 34^0/7^–36^6/7^ weeks of gestational age (GA)] infants have significantly more medical problems, resulting in markedly increased hospital costs compared with full term infants (FT: 39^0/7^–40^6/7^ GA) (Wang et al., 2004). Data from a population study from 2006 to 2014 in the United States showed that LPT birth rate was 6%, while early term (ET: 37^0/7^–38^6/7^ GA) rate was 26.9% (Richards et al., 2016). Late preterm and early term birth are associated with adverse neonatal outcomes, such as higher incidence of respiratory distress syndrome, temperature instability, hypoglycemia, hyperbilirubinemia, apnea, feeding problems, as well as higher rates of re-hospitalization and a two-fold increase in Sudden Infant Death Syndrome (SIDS) (Thompson and Mitchell, 2006; Loftin et al., 2010; Adamkin, 2013). Limited sleep state regulation, frequent episodes of apneas, periodic breathing, altered pulmonary function, bradycardia, and diminished autonomic control of heart rate (HR) have been documented in these populations (Hunt, 2006; Scher et al., 2011; McEvoy et al., 2013; Lucchini et al., 2018).

Starting from a concept introduced by the new field of Network Physiology, the human organism can be viewed as a network of integrated and interacting physiological systems (Ivanov and Bartsch, 2014; Ivanov et al., 2016). Thus, given the described adverse conditions related to imbalances of both cardiac and respiratory systems, investigation of risks associated with late prematurity should include a focus on the dynamic interaction in the cardiorespiratory network. Regulation and autonomic control of respiratory and cardiovascular interactions are crucial for the maintenance of homeostasis during sleep (Harper et al., 1988). In adults, many studies have shown evidence that cardiorespiratory imbalance is associated with obstructive sleep apnea and heart failure, resulting in higher sympathetic tone and potentially ultimately triggering life-threatening events (Harper et al., 2012). Similarly, it has been reported that nocturnal perturbations of cardiac and respiratory systems in newborns play a crucial contributory role in SIDS (Schechtman et al., 1991). Despite the high clinical relevance there is a paucity of research data about the mechanisms related to cardiorespiratory interactions early in life when the primary control systems are still developing.

Many approaches have been proposed in the past to address the complex interaction of the cardiorespiratory system, from simple time and frequency domain measures (Horne, 2014) to more complex ones, such as those based on information theory (Frasch et al., 2007; Bartsch et al., 2012; Penzel et al., 2016). These, in particular Transfer Entropy (*TE*), are progressively gaining interest as model-free approaches which quantify directional interaction between subsystems and are thus sensitive to both linear and non-linear interactions. In prior publications the existence of several co-existing forms of cardio-respiratory coupling (Bartsch et al., 2014) has been shown, and our group has also addressed this topic analyzing cardiorespiratory interaction with regards to entropy and phase locking (Lucchini et al., 2017, 2018). In the current report, we propose a new application of *TE* measure to provide an estimation of information transfer between the cardiac and the respiratory system at various lags. The focus on the timing of such interactions will augment descriptive approaches for assessing cardiorespiratory interplay at various time scales.

Specifically, we are interested in characterizing such system crosstalk in a population of LPT, ET, and FT infants. This investigation aims to provide insight into developing control systems involved in the cardiorespiratory regulation and how prematurity affects this complex interaction. This could inform interventions aimed at reducing risk for morbidities and mortality in this population.

## Materials and Methods

### Lagged Transfer Entropy

For our proposed framework, we modeled a dynamical system composed of two interacting sub-systems (*M* = 2), whose visited states can be represented by discrete-time stationary stochastic processes, namely *X* and *Y*. In this context, *TE* aims at evaluating the information transfer by the past states of the process *X* about the present of the process *Y*, that is not already provided by the past states of *Y* (directionality *X→Y*) and vice versa (directionality *X→Y*) (Schreiber, 2000).

We define *x*_*n*_, *y*_*n*_ as the stochastic variables representing the present states of the processes *X* and *Y* at a given time point *n*, with *n* < *N* and *N* = length of the signals, and *x_1:n_*_–1_, *y_1:n_*_–1_ the vectors of their respective past states.

Transfer Entropy is defined accordingly to Eq. 1:

(1) $${\mathrm{TE}}_{X\to Y}=\sum p\,\left(y_{1:n},x_{1:n-1}\right)\,\log\,\frac{p\,\left(y_{n}|x_{1:n-1},y_{1:n-1}\right)}{p\,\left(y_{n}|y_{1:n-1}\right)}$$

where the sum incorporates all states visited by the subsystems.

Similarly, the formulation of *TE* can be expressed in terms of the difference between two Conditional Entropy (*CE*) terms as shown in Eq. 2:

(2) $${\mathrm{TE}}_{X\to Y}=H\,\left(y_{n}|y_{1:n-1}\right)-H\,\left(y_{n}|x_{1:n-1},y_{1:n-1}\right)$$

The previously reported *TE* formulations encompassed an aggregate measure of information transfer which is not candidate-specific, where candidate refers to one of the elements contained in the vectors employed to reconstruct the past of processes *X* or *Y* at the instant *n* defined as *x_1:n_*_–1_ and *y_1:n_*_–1_.

In this work, to disambiguate the contribution of different candidates toward the estimate of *TE*, we employed the approach described in Faes et al. (2014). Given the *TE* formulation expressed in Eq. 2, we computed *TE* based on a sequential procedure for non-uniform conditioning, where the conditioning vector is updated progressively by selecting the candidate which reduced the most uncertainty in explaining the target variable. The initial set of candidates was defined including a predefined maximum number of past states, i.e., Ω = {*X*_*n*–1_, *X*_*n*–2_,.., *X*_*n*–_*_*Lmax*_*, *Y*_*n*–1_, *Y*_*n*–2_,.., *Y*_*n*–_*_*Lmax*_*}. In this work, the maximum number of candidates (*L*_*max*_) was set equal to 10. Candidates were progressively selected among the elements of Ω as described in Faes et al. (2014). Once the selection procedure has terminated, the vectors of candidates for both *X* and *Y* processes were produced and defined as *Vk* = [*V_*k*_^*X*^*, *V_*k*_^*Y*^*]. Thus, they were suitable to be employed as conditioning vectors for further *TE* estimations. Given the reported notation, Eq. 2 can be rewritten employing conditioning vector formulation as reported in Eq. 3:

(3) $${\mathrm{TE}}_{X\to Y}=H\,\left(y_{n},V_{k}^{Y}\right)-H\,\left(V_{k}^{Y}\right)-H\,\left(y_{n},V_{k}\right)+H\,\left(V_{k}\right)$$

The final step to *TE* estimation relied on the computation of probability density functions to approximate the interrelationship between *X* and *Y*, based uniquely on single realizations of the two processes. The practical estimation of terms in Eq. 3 was based on the previously defined embedding vector (*V*_*k*_) and it employed a Nearest Neighbor (*NN*) estimator. The combination of non-uniform embedding and *NN* estimator (*NN NUE*) has been reported to be optimal for *TE* estimation (Kugiumtzis, 2013). Furthermore, the statistical significance of computed *TE* was assessed using surrogate data implemented by time shift procedure. In this analysis, the number of employed surrogate series was equal to 100, with a maximum allowed time shift of 20 samples. The significance threshold was set above the 95th percentile of the surrogate series distribution.

Transfer Entropy has been usually employed for a global measure of information transfer between time series. This work proposes a novel approach toward the quantification of the instantaneous and delayed effects among two interacting systems based on *TE* notion. This application lies its foundations on the previously described *TE* implementation, yet it considers several lagged versions of the original series (Pini et al., 2019).

In order to quantify *TE_*X*→*Y*_* at a lag value (*τ*) equal to one, the target series was shifted forward of one sample so that *x*(*n*) was aligned with *y*(*n–1*). The lagged version of TE proposed in this approach aims at quantifying the information provided by the past of *X* on the shifted portion of the process *Y*, that is not already provided by the past of *Y* as reported in Eq. 4:

(4) $${\mathrm{TE}}_{X\to Y}=\sum p\,\left(y_{1-\tau :n-\tau },x_{1:n-1}\right)\,\log\,\frac{p\,\left(y_{n-\tau }|x_{1:n-1},y_{1-\tau :n-1-\tau }\right)}{p\,\left(y_{n-\tau }|y_{1-\tau :n-1-\tau }\right)}$$

The underlying idea is to quantify the source series effects on the target and the instantaneous and delayed effects between the two processes. The previously described computational implementations for *TE* are again employed for this proposed lagged version. For this analysis the maximum lag between *X* and *Y* series was set to 15. The statistical significance of *TE* estimations for each lag was tested with surrogate data, as previously described.

### Validation

To provide validation of the proposed methodology, lagged TE was computed based on a dynamical system composed by *M* = 2 stochastic dynamic subsystems, namely *X* and *Y*, defined by Eq. 5:

(5) $$\begin{array}{c} X_{n}=a_{1}\,X_{n-1}+a_{2}\,X_{n-2}+0.07\,Y_{n-1}+U_{n}\\ Y_{n}=C_{1}\,X_{n-1}+C_{2}\,X_{n-2}+C_{3}\,X_{n-5}+V_{n} \end{array}$$

where *U*_*n*_ and *V*_*n*_ are independent white noises with zero mean and unitary variance. The autoregressive parameters *a*_1_, *a*_2_, *C*_1_, *C*_2_, and *C*_3_ were set as described in Faes et al. (2014). Process *X* simulates a self-sustained rhythm with a characteristic frequency centered at *f* = 0.1 Hz. Parameters and delay settings were chosen to simulate two different types of interaction: one which is, lasting and strong taking into consideration the directionality *X→Y*, the other which is transient and weak for *Y→X*. The simulated series length was set to *N* = 300 points, and the total number of generated series was equal to 100 for each lag, ranging from the unlagged version (lag = 0) of the series to their maximum lagged version (lag = 15). As previously described in the Methods section, the statistical significance of TE estimations for each lag was tested with surrogate data. Two one-way ANOVAs were performed to test the interaction between the fixed factor lag and each dependent variable (*TE_*X*→*Y*_* or *TE_*Y*→*X*_*).

### Experimental Protocol and Data Preprocessing

For this analysis our dataset included 268 infants born at the Morgan Stanley Children’s Hospital of New York between 35^0/7^ and 40^6/7^ weeks of GA. No participating newborn was admitted to the Neonatal Intensive Care Unit, and there was no evidence of major illness, genetic disorders. Also, there was no past/present medicated/non-medicated psychiatric complaints in the mothers. A minimum Apgar score of 8 after 5 min of life was required. Mothers signed informed consent forms prior to enrollment in the study. The Institutional Review Boards of the New York State Psychiatric Institute and Columbia University Medical Center approved all consent and data acquisition procedures.

Subjects who met inclusion criteria were tested 12–84 h after birth (mean and standard deviation of hours of life = 48 ± 12 h). Infants were grouped based on GA: LPT (*N* = 67), ET (*N* = 91), and FT (*N* = 110). Within ∼30 min after feeding, infants were put supine to sleep and data acquisition lasted 10 min. ECG and respiration signals were acquired at 500 Hz and 200 Hz, respectively. ECG was recorded with three leads, placed on the infant’s chest (left abdomen, left and right scapula) and the signal was amplified and recorded using the DATAQ Instruments ECG system (Medelex, New York City, NY, United States). A respiratory inductance belt (Ambulatory Monitoring Inc., Ardsley, NY, United States) was placed around the infant’s abdomen to measure the respiration signal. Sleep states were classified into active sleep (AS) and quiet sleep (QS) based on respiratory variability and confirmed by behavioral codes entered throughout the study to determine when infants were awake, crying, or fussy (Stefanski et al., 1984; Isler et al., 2016).

The R peaks were detected on the ECG with proprietary software (Gmark, Ledano Solutions) based on the Pan–Tompkins algorithm and subsequently checked by visual inspection. The respiration signal was bandpass filtered (0.05–3.5 Hz). The thresholds of acceptance for RR interval were set as 0.3–0.667 s, with an absolute variation between consecutive RR intervals of 10%, while for respiration thresholds were 0.5–2.5 s (absolute change 40%). Segments with more than 5% rejected samples were discarded from further analysis. The RR series was then defined so that *RR*(*n*), was the time interval between the n-*th* R peak and the successive one at a time (*n* + 1)-*th*. Similarly, the n-*th* sample of resampled respiration series *RESP*(*n*) was obtained by resampling the original respiration series at the onset of the n-*th* R peak which coincides with the time previously defined for *RR*(*n*). Within the same sleep state, segments of 300 consecutive RR intervals (*RR*) and 300 respiration samples (*RESP*) were identified. The resulting series, *RR*(*n*) and *RESP*(*n*) with *n* = 1, …, 300, were normalized to zero mean and unit variance to be employed for further analysis. The segments length was chosen based on previous studies, reporting 300 samples as appropriate for a reliable *TE* estimation as fulfilling the requirement of stationarity (Faes et al., 2014; Lucchini et al., 2017). The described preprocessing pipeline was necessary to avoid potential bias in the further analysis. Specifically, the effect of non-stationarities over entropy measures and estimators due to artifacts has been extensively shown in Xiong et al. (2017).

The total number of analyzed segments was 661, 392 in AS and 269 in QS. The MuTE toolbox was employed for computing Transfer Entropy (Montalto et al., 2014).

Two-way ANOVAs tested the effect of fixed factors lag (0:15) and GA (LPT, ET, FT) on *TE_*RR*→*RESP*_* and *TE_*RESP*→*RR*_* for each sleep state. Sex, mode of delivery (MoD), and hours of life (HoL) were included in the statistical model as covariates. Significance for fixed factors as well as their interactions were tested. A series of *post hoc* tests were performed: simple main effects and specific group differences. Statistical analysis was conducted with IBM SPSS Statistics for Windows, Version 25.0. Armonk, NY: IBM Corp.

## Results

### Validation Data

**FIGURE 1:**
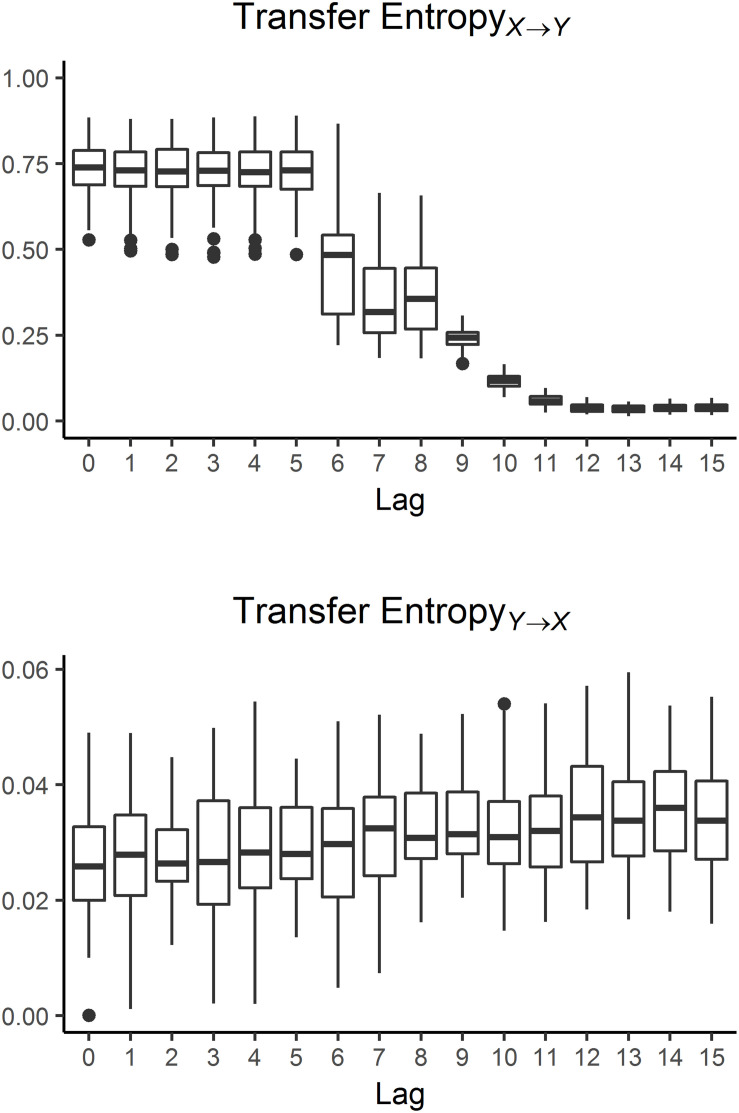
TE estimates for the directionality *X→Y* **(top)** and *Y→X* **(bottom)** computed on simulate dataset. *TE_*X*→*Y*_* exhibit a marked influence as lags are progressively increasing. On the contrary, no influence of lags over *TE_*Y*→*X*_* is detected.

The top panel of Figure 1 shows *TE_*X*→*Y*_* as a function of lag, computed on a simulated dataset. The behavior of *TE_*X*→*Y*_* is in accordance with the simulated interaction between subsystems *X* and *Y*. Specifically, *TE_*X*→*Y*_* exhibits a strong and stable influence of process *X* over process *Y* for lags 0 to 5, where the information transfer between the two series is expected to be maximum given that *X*_*n*–1_, *X*_*n*–2_, and *X*_*n*–5_ are effectively contributing to modulate the target series *Y*. The rapid reduction in TE at lags equal to 6 and 9 are consistent with the set delays. Specifically, at lag 6, the past state *X*_*n*–5_ of process *X* cannot be included in the conditioning vector anymore, given the chosen maximum candidate delay *L* = 10. Accordingly, a net decrease in *TE* is noticed when passing from lag = 5 to lag = 6. Analogous reasoning applies when moving from lag = 8 to lag = 9. Lastly, from lag = 10 on, the mutual influence in the directionality *X→Y* becomes negligible given the loss of interaction between the two sub-systems, thus resulting in *TE* estimates close to zero. Statistical analysis reveals a significant effect of lag over *TE_*X*→*Y*_* (*p*-value < 0.001). Bonferroni *post hoc* tests showed significant differences of lag 0–5 vs. lag 6–15, lag 11–15 vs. lag 0–10, and lags 6, 7, 8, 9, 10 are significantly different from each other.

**FIGURE 2:**
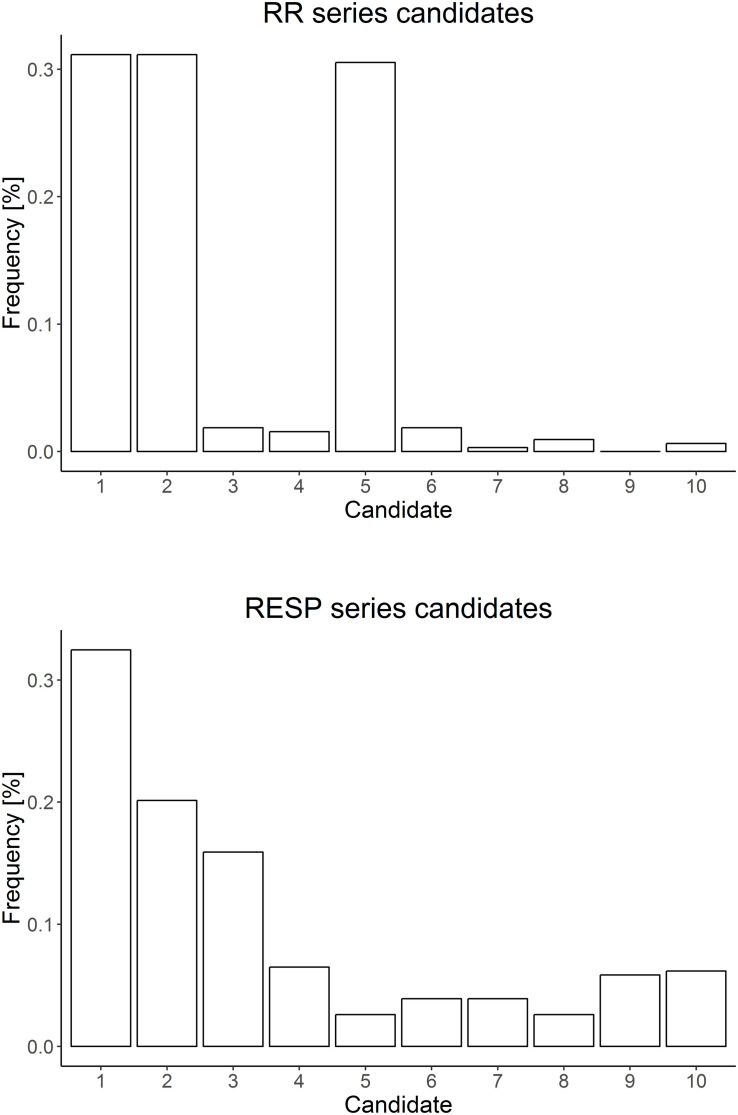
*X* **(top)** and *Y* **(bottom)** series candidate distributions employed for TE computations considering lag = 0. RR series candidate distribution shows a proper selection of the simulated delays. On the other hand, RESP candidate distribution reflects the weak modulation effect of series *Y* on *X.*

Distribution of selected *RR* candidates included in the conditioning vector *V_*k*_^*X*^* referring to *TE* at lag = 0 is displayed in the top panel of Figure 2. The frequency of selected candidates is in accordance with the simulated interaction delay between the two series, namely *X*_*n*–1_, *X*_*n*–2_, and *X*_*n*–5_.

With regard to *TE_*Y*→*X*_*, values are stable across all the lags and noticeable lower when compared with estimates for *TE_*X*→*Y*_*, as presented in the bottom panel of Figure 1. The absence of any *TE_*Y*→*X*_* significant differences by lags reflects the weak and transient influence of the information transfer for this directionality. Uniformly, only *Y*_*n*–1_ results the preferred candidate as it effectively contributing to modulate the target series *X* as a standalone past sample of series *Y*, as shown in the bottom panel of Figure 2.

### Cardiorespiratory Data

Using the same approach described in see section “Validation” for the simulated dataset, *TE* estimations across lag and GA, as well as *RR* and *RESP* candidate distributions were computed. Additionally, the statistical significance of *TE* estimations for each lag was tested employing surrogate data.

**FIGURE 3:**
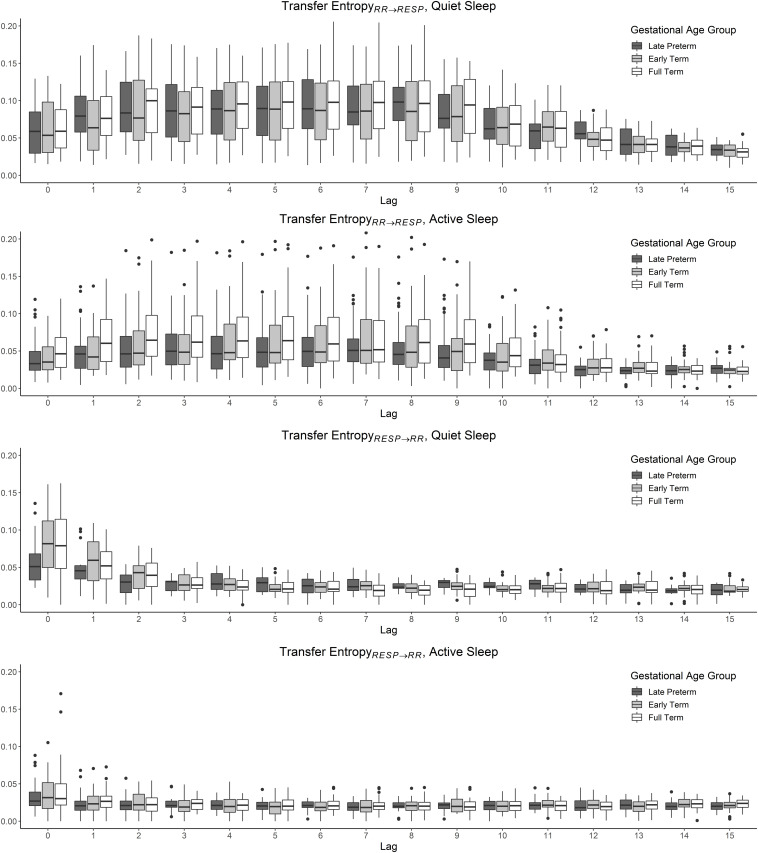
TE estimates computed for cardiorespiratory dataset considering the three GA groups: LPT, ET, and FT, separately for QS and AS. The behavior of *TE_*RR*→*RESP*_* resembles a longer and stable effect of RR modulation over RESP. On the contrary, *TE_*RESP*→*RR*_* exhibit a transient and rapidly decreasing interaction between the subsystems.

The subsystems’ interaction for the directionality *RR→RESP* exhibited a long-lasting and steady effect of cardiac system modulation over the respiratory system, as shown in the top two panels of Figure 3. No differences were found when comparing *TE_*RR*→*RESP*_* across lags in the interval 0–9, consistently for both QS and AS. Analogous behavior was displayed in the interval 10–15. In contrast, *post hoc* tests revealed significant differences comparing each lag in the interval 0–9 vs. 10–15. Significant GA group differences were found when considering estimates of *TE_*RR*→*RESP*_* in AS only. Specifically, we observed an average increase in information flow across GA. This was confirmed by the *post hoc* test comparing LPT vs. ET (*p* < 0.001), LPT vs. FT (*p* < 0.001), and ET vs. FT (*p* < 0.001). *RR* candidates employed in estimating *TE_*RR*→*RESP*_* at lag = 0 exhibit a similar frequency of selection for both QS and AS. Moreover, when investigating the role of GA for candidate frequency of selection, no differences are found across age.

**FIGURE 4:**
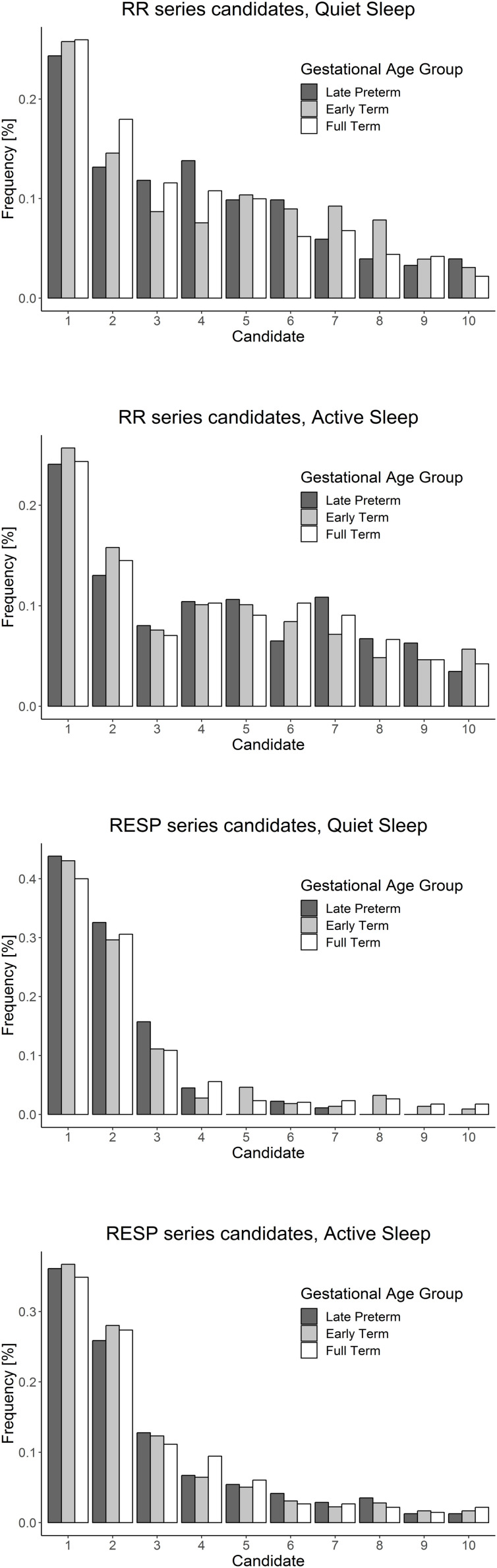
RR **(top two panels)** and RESP **(bottom two panels)** series candidate distributions employed for TE computations of cardiorespiratory data. RR candidates are selected uniformly among all possible candidates. On the opposite considering the respiratory signal, the first two possible RESP candidates are selected with higher probability.

In contrast to what previously reported for *TE_*RR*→*RESP*_*, *TE_*RESP*→*RR*_* exhibited, the current study demonstrated a decrease in information transfer from *RESP* to *RR* as lags were progressively increasing in both QS and AS states. As confirmed by statistical analysis and shown in the two bottom panels of Figure 3, no significant differences among lags were found for lags >3. Given this finding we limited the analysis to a restricted poll of lags, specifically 0–3, with the aim of avoiding over-representing similar class distributions in the successive analysis. The statistical analysis performed on the subgroups of lags for *TE_*RESP*→*RR*_* showed a significant effect of lag as a fixed factor. Specifically, each lag was consistently different from each other for both QS and AS. Analyses among GA groups report no differences for *TE_*RESP*→*RR*_* in AS. Significant differences were evident in QS. A summary of statistical results is reported in Table 1. The candidate analysis reported a behavior characterized by a prevalent selection of *Y_*n*–1_* and *Y_*n*–2_* candidates for both sleep states as displayed in Figure 4.

**TABLE 1:** Results of two-way ANOVA tests on cardiorespiratory data.

Directionality	Fixed factor	Quiet sleep	Active sleep
*RR→RESP*	Lag	<0.001	<0.001
	GA	0.120	<0.001
	Lag*GA	1.000	0.897
*RESP→RR*	Lag	<0.001	<0.001
	GA	0.050	0.183
	Lag*GA	0.627	0.953

Given the differences of *TE_*RR*→*RESP*_* and *TE_*RESP*→*RR*_* as a function of GA, we decided to investigate the role of breathing rate for our model. The rationale for investigating breathing frequency was based on previous studies showing differences by sleep state (higher breathing rate in AS) but not by GA (Lucchini et al., 2018). Thus, we hypothesized that breathing rate is partly mediating the interaction between sleep states and TE.

In this analysis, we first tested (Sobel test) a model having sleep states as the independent variable (IV), *TE* as the dependent variable (DV) and breathing frequency as a mediator (M). Partial mediation analysis quantifies the decrease in correlation strength among two factors, once a specific mediator is introduced in the model. Specifically, when considering *TE_*RR*→*RESP*_* as DV, the correlation between IV and DV was significant (*p*-value < 0.001) and the mediation effect of breathing frequency was equal to 11%, similar results were obtained considering *TE_*RESP*→*RR*_* as DV (*p*-value = 0.001 and 14%). On the other hand, when testing GA as the DV, no significant mediation was reported in either TE directionality.

## Discussion

The goal of this study was to analyze the maturation of the cardiorespiratory networks in terms of information flow dynamics in a population of newborns during sleep. Gaining insight on such interactions attains the potential for assessing individual differences in neonatal control mechanisms and vulnerability for the reported higher morbidity and mortality rates in LPT and ET newborns (Richards et al., 2016). Investigation of the neurophysiological mechanisms responsible for cardiorespiratory regulation is challenging, due to their intrinsic complexity and the requirement to employ non-invasive monitoring. Quantitative analysis of cardiorespiratory interactions in the newborn nursery represents a valuable investigation tool. Moreover, the derived parameters provide a window of opportunity to observe non-invasively the interaction between sympathetic and parasympathetic nervous systems and their capability to timely respond to internal and external challenges.

In this study, we propose *TE* as an optimal method to investigate the above-mentioned interaction (Kugiumtzis, 2013). The advantages of the proposed approach are multiple. Firstly, it is model-free, i.e., it does not require any *a priori* assumption regarding the systems generating the observed data. This is crucial in the neonatal context, given that control systems at birth are still developing and the typical cardiorespiratory models for adults thus cannot be applied. Even in the context of integrated system physiology in adult subjects, general models cannot be directly employed but often need to be modified and adapted in accordance with the observed dynamics. Secondly, TE is a measure encompassing the dynamics of information transfer and thus it provides an indication of directionality (Schreiber, 2000). This is particularly important as it is well documented that respiratory and cardiovascular rhythms influence each other due to central as well as peripheral nervous mechanisms of interaction (Bartsch et al., 2015). Despite these two important advantages there is one intrinsic limitation of traditional TE techniques, namely the reliance on a single global measure of information. Thus, TE lacks information about different time scales of information flow across subsystems. Gaining an understanding of the different time scales at which the vagal and sympathetic mechanisms operate would augment the description of ANS modulating action over cardiorespiratory interaction (Faes et al., 2014).

To specifically address this issue, this paper proposes an extension of traditional TE formulation. It complements the estimates of magnitude and directionality of information flow with that of timing between the two coupled processes. Accordingly, TE was calculated on several lagged versions of the original series.

To provide validation for the proposed methodology, computation of lagged TE was performed based on a dynamical system composed of two stochastic subsystems simulating two different types of interaction: a lasting and strong one considering the directionality X→Y, paired with a transient and weak one for Y→X. The validation procedure performed on simulated data confirmed the ability of lagged TE to track the information transfer at different time scales.

The resulting application of lagged TE on neonatal cardiorespiratory data showed two distinct interaction profiles as a function of directionality: a fast and quickly decaying information transfer from RESP to RR, and a slower but more stable transfer from RR to RESP. Convergent findings with regard to the directionality from RESP to RR were obtained by Faes et al. (2014), showing that the fast information flow from breathing to HR is associated with the respiratory sinus arrhythmia. Furthermore, the timing of activation of the information flow profile is comparable to the known latencies of activation for the sympathetic and parasympathetic arms of the nervous system. Specifically, the sympathetic branch intervenes on a slower time scale but its effect on the target system lasts longer whereas the parasympathetic has a punctate, yet rapidly vanishing action. Thus, the reported lagged TE dynamics might reflect that information transfer directionalities are driven by different autonomic branches of the ANS (Hoyer et al., 2005). This is relevant in the context of possible approaches for quantification of sympathetic activation. A state of sympathetic hyperactivity has been in fact reported as associated with an increase in cardiovascular morbidity and mortality (Brook and Julius, 2000; Nakamura et al., 2016). Thus, while several heart rate variability parameters can assess parasympathetic activity, consensus about quantification of sympathetic nervous system activity is still pending.

Regarding the differences as a function of GA, we reported a significant decrease of information flow in LPT from RESP to RR in quiet sleep, and for LPT and ET a reduced information flow from RR to RESP in active sleep. Interestingly, there was no difference in the candidates selected for the reconstruction of the past states. In line with our findings, previous work addressing other forms of cardiorespiratory interaction in newborn population highlighted that the direction of coupling between cardiovascular and respiratory systems varies with age over the first 6 months of life, with a tendency to change from a nearly symmetric bidirectional interaction to primarily unidirectional mode from *RESP* to *RR* (Rosenblum et al., 2002; Lucchini et al., 2016). These findings strengthen the assumption that GA-related differences are due to intrinsic differences in the interactions between subsystems, given the reported candidate selection employed for the reconstruction of RR series past vector. The reported GA-related results constitute a plausible explanation for cardiorespiratory differences in the newborn period and might pave the way to a possible explanation for the increased risk of LPT and ET populations.

To extend these findings, we explored the role of breathing rate on the modulation of the information flow. Our partial correlation model confirmed the role of breathing rate as a mediator for the interaction between sleep states and TE, but not between GA and TE. These results mirror our previous findings regarding cardiorespiratory interactions. We previously reported the absence of a change in breathing frequency as a function of GA. However, a significant modification of directionality of the cardiorespiratory coupling had been observed (Lucchini et al., 2018).

One limitation of the presented investigation is the absence of arterial blood pressure (ABP) included in the model. The availability of this additional signal would lead to a more comprehensive investigation of the complex physiological interactions of the cardiovascular systems as a function of state and age (Xiao et al., 2005). Lastly, larger scale studies are needed to investigate neonatal ANS regulation in the context of diverse factors, e.g., ethnicity, socio-economic status, maternal conditions, psychosocial stressors.

In conclusion, the utilization of a lagged version of TE might lead to a novel approach to investigate physiologic networks, selectively assessing horizontal information transfer at different time scales. This particular investigation of the interaction between the cardiac and respiratory systems aimed at characterizing the different regulatory profiles of the two branches of the ANS and at ultimately providing an indication of altered patterns of physiological behavior. Findings presented in this paper are convergent with previous published findings (Frasch et al., 2007; Faes et al., 2014). The novel contribution of this study is the characterization of the dynamics of the cardiorespiratory network across sleep states and gestational ages. Ultimately, application of TE in assessment of network physiology affords an opportunity for early risk stratification in a high risk population (Bartsch et al., 2015). In the near future, we envision the application of TE methodology for the characterization of other interacting subsystems such as brain-brain and brain-heart to provide a more comprehensive picture of the complex mechanisms characterizing neonatal development.

## Data Availability Statement

The datasets generated for this study are available on request to the corresponding author.

## Ethics Statement

The studies involving human participants were reviewed and approved by the Institutional Review Boards of the New York State Psychiatric Institute and Columbia University Irving Medical Center. Written informed consent to participate in this study was provided by the participants’ legal guardian/next of kin.

## Author Contributions

NB and WF contributed to the conception and design of the study. NB carried out the recording of the data. ML and NP planned and carried out the simulations and analysis of data and took the lead in writing the manuscript. ML, NP, NB, MS, and WF contributed to the interpretation of the results. All authors provided critical feedback and helped shape the research, analysis and manuscript.

## Conflict of Interest

The authors declare that the research was conducted in the absence of any commercial or financial relationships that could be construed as a potential conflict of interest.
